# 

**Published:** 1902-04-15

**Authors:** 


### ANNOUNCEMENTS.

### THE ILLINOIS STATE DENTAL SOCIETY.

The Illinois State Dental Society will meet in annual session at Springfield, Ills., May 13th, 14th,, 15th. Practical papers and clinics by the best men in the profession will fill the entire time of the session.

### THE NORTHERN OHIO DENTAL SOCIETY.

The Northern Ohio Dental Society will hold its forty-third annual meeting at Cleveland, June 9th, 10th and 11th. Fourteen papers are on the program and fourteen clinics scheduled. A foot note on the program reads as follows: "Dear Doctor: If you have resolved that you will not attend the next meeting of the N. O. D. A., don't read this program, for it means a broken resolution if you do."

### SOUTH CAROLINA STATE ASSOCIATION AND THE STATE BOARD OF EXAMINERS.

The thirty-second annual meeting of the South Carolina State Association and the State Board of Examiners will be held in Charleston, May 13, 1902. All dentists in the State are invited to be present, and visiting dentists from other States will be cordially welcomed. This will be an excellent time to visit the historic "city by the sea," and also the exposition.

### THE AMERICAN SOCIETY OF ORTHODONTISTS.

The American Society of Orthodontists will hold its second annual session in Philadelphia this year on the 8th, 9th and 10th of October. A complete program is in preparation and will be issued in due time.

